# Drug–target affinity prediction with extended graph learning-convolutional networks

**DOI:** 10.1186/s12859-024-05698-6

**Published:** 2024-02-16

**Authors:** Haiou Qi, Ting Yu, Wenwen Yu, Chenxi Liu

**Affiliations:** 1 Nursing Department, Sir Run Run Shaw Hospital, Zhejiang University School of Medicine, Hangzhou, 310016 China; 2 Operating Room Department, Sir Run Run Shaw Hospital, Zhejiang University School of Medicine, Hangzhou, 310016 China; 3 School of Artificial Intelligence and Automation, Huazhong University of Science and Technology, Wuhan, 430074 China; 4 School of Medicine and Health Management, Tongji Medical School, Huazhong University of Science and Technology, Wuhan, 430030 China

**Keywords:** Drug–target affinity prediction, Deep learning, Drug discovery, Graph learning-convolutional networks

## Abstract

**Background:**

High-performance computing plays a pivotal role in computer-aided drug design, a field that holds significant promise in pharmaceutical research. The prediction of drug–target affinity (DTA) is a crucial stage in this process, potentially accelerating drug development through rapid and extensive preliminary compound screening, while also minimizing resource utilization and costs. Recently, the incorporation of deep learning into DTA prediction and the enhancement of its accuracy have emerged as key areas of interest in the research community. Drugs and targets can be characterized through various methods, including structure-based, sequence-based, and graph-based representations. Despite the progress in structure and sequence-based techniques, they tend to provide limited feature information. Conversely, graph-based approaches have risen to prominence, attracting considerable attention for their comprehensive data representation capabilities. Recent studies have focused on constructing protein and drug molecular graphs using sequences and SMILES, subsequently deriving representations through graph neural networks. However, these graph-based approaches are limited by the use of a *fixed adjacent matrix* of protein and drug molecular graphs for graph convolution. This limitation restricts the learning of comprehensive feature representations from intricate compound and protein structures, consequently impeding the full potential of graph-based feature representation in DTA prediction. This, in turn, significantly impacts the models’ generalization capabilities in the complex realm of drug discovery.

**Results:**

To tackle these challenges, we introduce GLCN-DTA, a model specifically designed for proficiency in DTA tasks. GLCN-DTA innovatively integrates a graph learning module into the existing graph architecture. This module is designed to learn a *soft adjacent matrix*, which effectively and efficiently refines the contextual structure of protein and drug molecular graphs. This advancement allows for learning richer structural information from protein and drug molecular graphs via graph convolution, specifically tailored for DTA tasks, compared to the conventional fixed adjacent matrix approach. A series of experiments have been conducted to validate the efficacy of the proposed GLCN-DTA method across diverse scenarios. The results demonstrate that GLCN-DTA possesses advantages in terms of robustness and high accuracy.

**Conclusions:**

The proposed GLCN-DTA model enhances DTA prediction performance by introducing a novel framework that synergizes graph learning operations with graph convolution operations, thereby achieving richer representations. GLCN-DTA does not distinguish between different protein classifications, including structurally ordered and intrinsically disordered proteins, focusing instead on improving feature representation. Therefore, its applicability scope may be more effective in scenarios involving structurally ordered proteins, while potentially being limited in contexts with intrinsically disordered proteins.

## Background

With the U.S. Food and Drug Administration (FDA) approval taking up to 17 years and costing $2.6 billion [1–5], drug development faces challenges, particularly under strict market scrutiny [6]. Drug repurposing and repositioning have emerged as key strategies to expedite and economize drug development [7], prompting a research focus on accurate prediction of drug–target affinity (DTA) [8–12]. The ability to predict binding interactions between small molecules and targets can significantly streamline the process of identifying lead compounds, thus expediting drug research and development.

Traditional methods based on biological experiments, e.g., high-throughput screening experiments, can be effective at predicting DTA, but they are too cumbersome and demand substantial amounts of money and time [13]. Meanwhile, the vast array of drug-like compounds, estimated in the millions [14], along with numerous potential targets, poses a significant challenge to the practical application of this technology. The expansion of bioinformatics databases in recent years has resulted in a substantial collection of biological experimental data, paving the way for the advent of computational approaches [5].

**Fig. 1:**
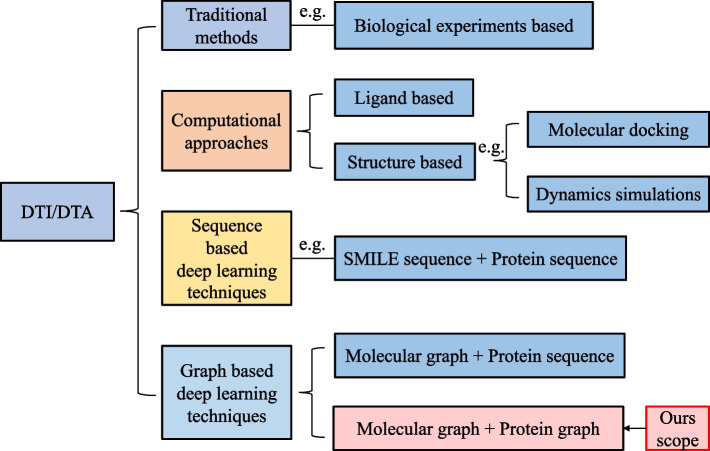
Summary of representative methods relevant to DTI and DTA prediction. Our methods apply to the scope of graph-based deep learning methods for DTA prediction

Existing computational approaches are roughly categorized into two types: ligand-based and structure-based approaches, as shown in Fig. 1. Ligand-based methods [15] are based on the concept that ligands with similar chemical properties are likely to have similar biological functions and bind to similar target proteins [16–19]. This approach focuses on the information about the ligand rather than the target protein’s structure. However, when the number of available ligands is low, the accuracy of predictions decreases. Structure-based methods, like docking simulations [20], use the 3D structures of drugs and target proteins to predict how well they bind. This is done using molecular docking and dynamics simulations [21–24]. While this method is accurate, it tends to be time-consuming. For proteins with available structural and site information, detailed simulations using molecular simulation and docking can yield highly precise results. However, many proteins lack such structural data, leading to molecular docking and dynamics simulations inapplicable. Sequence-based methods are an alternative method to predict their drug molecule binding affinity when there is lacking structural information on proteins. But the complexity of protein and small molecule structures makes accurately describing and extracting the features of targets and drugs a challenging aspect of affinity prediction. This challenge has become a focal point in computer-aided medicine research, particularly with the surge in deep learning applications over the last decade.

Currently, sequence-based techniques are leading the way in computational biology. The development of deep learning has inspired a wave of early studies to employ neural network architectures for the extraction of features from protein sequences and drug molecules SMILES (simplified molecular input line entry specification) [25], thereby more effectively learning about the potential characteristics of target proteins and drugs. Depending on the objectives, sequence-based methodologies are classified into two groups: drug–target interaction (DTI) prediction and DTA prediction. Specifically, within any given drug–target pair, deep learning algorithms are used to extract distinct representations of the drug and target. These are then concatenated into one comprehensive vector that is used for the final predictive analysis. DTI is a binary classification task used for determining whether the drug can bind to the target or not. For instance, TransformerCPI [26] employs a transformer-based neural network [27] to analyze sequence data for predicting drug–target interaction. NeoDTI [28] combines data from various heterogeneous networks, utilizing representations of drugs and targets that preserve their topological features for predicting interactions. Hu et al. [29] introduced a CNN-based technique for drug–target interaction prediction, incorporating both 1D and 2D structural descriptors of the drug and the protein’s sequence as inputs to the network. Ezzat et al. [30] further refine the prediction accuracy by using decision tree methods and kernel ridge regression for feature dimension reduction and ensemble learning.

DTA prediction, distinct from DTI prediction, aims to accurately predict the precise binding affinity between a drug and a target. This challenge, commonly seen as a regression task, has become increasingly interesting in recent years [31–33]. At present, various approaches have yielded impressive results in the realm of affinity prediction. For example, DeepDTA [34] utilizes a pair of convolutional neural networks (CNNs) to separately extract features from the drug and protein, later combining these features for predicting affinity. Additionally, DeepDTA has gathered previous research data to develop two benchmark datasets, where drugs are denoted in SMILES format and proteins by their sequences. This approach, involving two tailored convolution networks for molecule and protein representation, has shown effective results in the benchmarks. WideDTA [35], an advancement of DeepDTA, integrated Live Max Common Substructure (LMCS) and Protein Motifs and Domains (PDM) into its structure. It uses four separate convolutional neural networks (CNNs) to encode these components into four unique representations. Huang et al. [36] introduced an innovative fingerprint feature vector for molecules, and utilized a Pseudo Substitution Matrix Representation (Pseudo-SMR) to depict protein sequences, aimed at enhancing drug–target interaction predictions. DeepPurpose [37] uses two specialized encoders for SMILES and sequence representation, combining the functionalities of CNN, RNN (recurrent neural network), and Transformer structures. For molecule representation, molecular fingerprints serve as a common technique, where the structural details of a molecule are encoded into strings or binary digits. Techniques such as extended connectivity fingerprints [38], atom environment descriptors (MOLPRINT2D) [39], and molecular access system keys (MACCS) [40] are examples of this approach. Altae-Tran et al. [41] detailed methods for learning significant small-molecule representations in scenarios where data is limited. Although sequence-based approaches have seen successes, they overlook the topological aspects of drug and protein molecules. Relying solely on CNN technology falls short in fully capturing the sequence details of drugs and proteins. Acknowledging the interactions between atoms and assigning varying weights and attention to them automatically is crucial.

Recently, graph neural networks (GNNs) [42] have gained recent prominence in bioinformatics for their ability to efficiently process non-Euclidean spatial data [5]. Utilizing the intrinsic qualities of nodes and their relational dynamics, GNNs effectively extract key feature information, essential for accurately identifying and predicting vertices or edges. Given the intricate correlation and diversity in biological data, molecular graphs aptly represent biological details, especially in terms of molecular structure and inter-molecular functional relations. GNN models stand out in pinpointing molecular features by assimilating information from neighboring nodes to understand both local and overarching network details. The Graph Convolutional Network (GCN) [43] and Graph Attention Network (GAT) [44] are commonly used models within the GNN framework, and their application in computer-aided drug design is progressively growing, e.g., graph-based DTA. GraphDTA [45] integrated GNN into DTA prediction by creating molecule graphs where atoms serve as nodes and bonds as edges, effectively representing drug molecules. In this model, CNNs are employed to extract high-level representations from protein sequences, while GNN models are applied to the molecular graphs, enhancing the performance of DTA predictions. MCN-CPI [46] and PADME [47] similarly construct graphs for molecule depiction and utilize GNNs for feature extraction in DTA prediction. The success of these methods underscores the effectiveness of GNNs in accurately characterizing small molecules.

Although these methods are adept at forming graphs to represent molecules and explore their structural properties, they tend to neglect the structural information embedded within protein sequences. In recent developments, various graph-based works have focused on predicting drug–target interactions by employing both molecule graphs and protein graphs, as opposed to using proteins’ primary sequences. For example, DGraphDTA [48] converts protein sequences into contact maps for graph construction. Concurrently, drug molecules are transformed from SMILES to molecular graphs using RDKit [49]. Both sets of graphs are then fed into a GNN to aid in richer feature extraction. DGraphDTA’s reliance on extensive database searches, especially during sequence alignment, results in lengthy processing times and reduced prediction accuracy. To combat this, WGNN-DTA [50] integrates evolutionary scale modeling (ESM) [51] for generating protein graphs, thereby streamlining the prediction process and yielding superior results. Additionally, DGraphDTA’s limitation in capturing target representation alterations due to DTIs, a result of its training on a limited protein dataset, is addressed by the newly proposed Graph Early Fusion Affinity (GEFA) [52]. GEFA transforms drug molecule graphs into protein graphs, utilizing an attention mechanism for more effective learning and prediction. To broaden the range of features derived from drug and protein graphs, STAMP-DPI [53] adopts Mol2vec [54] to analyze inter-drug relationships. Concurrently, it uses a pre-trained BERT [55] model to extract embedded representations from the large pool of unlabeled protein sequences provided by TAPE [56].

Utilizing graph-based methodologies, which focus on the topology and functionality of graphs, allows for the exploration of complex protein structure features, thereby increasing the accuracy and reliability of predictions. However, these graph-based approaches are limited by the use of a *fixed adjacent matrix* of protein and drug molecular graphs for graph convolution. This limitation restricts the learning of comprehensive feature representations from intricate compound and protein structures, consequently impeding the full potential of graph-based feature representation in DTA prediction. This, in turn, significantly impacts the models’ generalization capabilities in the complex realm of drug discovery.

To tackle these challenges, we introduce GLCN-DTA, a model specifically designed for proficiency in DTA tasks. GLCN-DTA innovatively integrates a graph learning module into the existing graph architecture. This module is designed to learn a *soft adjacent matrix*, which effectively and efficiently refines the contextual structure of protein and drug molecular graphs. This advancement allows for learning richer structural information from protein and drug molecular graphs via graph convolution, specifically tailored for DTA tasks, compared to the conventional fixed adjacent matrix approach. A series of experiments have been conducted to validate the efficacy of the proposed GLCN-DTA method across diverse scenarios. The results demonstrate that GLCN-DTA possesses advantages in terms of robustness and high accuracy. The proposed GLCN-DTA model enhances DTA prediction performance by introducing a novel framework that synergizes graph learning operations with graph convolution operations, thereby achieving richer representations. Note that our method, similar to other graph-based deep learning approaches [48, 50, 52, 53], primarily focuses on better expressing and extracting the characteristics of drugs and targets, fusing these features, and then predicting affinity. It does not consider the classification of proteins, nor does it differentiate between structurally ordered proteins and intrinsically disordered proteins. The focus is more on how to better represent features. Consequently, the applicability of our method might be limited, where it may perform better in scenarios involving structurally ordered proteins but could be limited when dealing with intrinsically disordered proteins.

## Methods

**Fig. 2:**
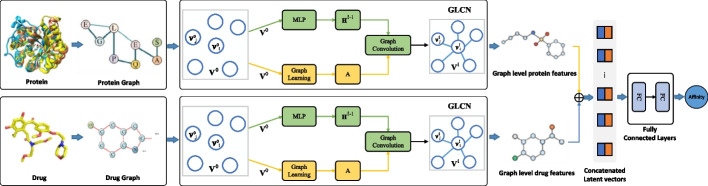
The architecture of GLCN-DTA. For protein sequences, protein graphs are built upon contact maps derived from their sequences. In the case of molecules, their SMILES representations are utilized as the foundation for graph construction. Once these two graph representations are established, they are processed through two separate graph learning-convolutional networks (GLCN) to extract their respective graph-level features. These representations are then concatenated to predict the affinity via fully connected layers. ${{\textbf {V}}}^{l}$ donates *node embedding* in the *l*-th graph convolution layer. ${{\textbf {H}}}^{l}$ represents *hidden features* in the *l*-th graph convolution layer. ${{\textbf {A}}}$ is *soft adjacent matrix*. $\oplus$ denotes concatenated operation

GLCN-DTA, a deep learning framework designed for graph-based DTA prediction, is depicted in Fig. 2 and consists of three key steps. (1) Generating drug molecule graph representation: GLCN-DTA inputs drug compounds in SMILES format and converts them into a drug graph that includes atoms and edges, on behalf of the drug’s natural properties. Inspired by existing graph network literature [57, 58], GLCN-DTA integrates an extended graph learning mechanism to existing graph convolutional architectures, formatting graph learning-convolutional networks (GLCN). The GLCN is designed to learn a *soft adjacency matrix* which refines the graph’s context, highlighting node relationships for DTA tasks and improving feature extraction through graph convolution. This graph convolution process efficiently uses the relationships from the graph learning module, spreading information between nodes in the graph. By default, GLCN-DTA uses a two-layer graph learning-convolutional network for extracting drug graph features. (2) Obtaining protein graph features: GLCN-DTA processes the protein sequence input and converts it into a contact map [48], using established methods such as Pconsc4. From this contact map, a protein graph is formed, applying a 0.5 threshold to create the initial adjacency matrix, as inspired by [48]. This contact map effectively represents the protein’s high-dimensional structure and is key to identifying interactions among protein residues. Typically, residues are deemed to be in contact if the Euclidean distance between any two atoms is below a certain threshold [59]. To extract detailed information from these protein graphs, a two-layer graph learning-convolutional network is also employed in this step by default, taking into account their contextual relationships. (3) Performing DTA prediction: GLCN-DTA utilizes the representations from both the molecule graph and the protein graph, combining them into a single, unified vector. This vector is then processed through a two-layer feed-forward network (FFN), enabling the method to arrive at the final binding prediction.

Significantly, GLCN-DTA sets itself apart from other graph-based methods such as DGraphDTA [48], WGNN-DTA [50], GEFA [52], and STAMP-DPI [53]. These methods typically require predefining specific edge types and node connectivities in the graph, or they implement thresholds on contact maps to establish a *fixed adjacency matrix*, which might introduce noise or inaccuracies in graph topology leading to aggregate useless and redundancy information between nodes. In contrast, GLCN-DTA leverages its graph learning module to automatically learn hidden node relationships, efficiently refining the graph’s structure and filtering useless nodes and being robust to complex structures. This process uncovers a *soft adjacency matrix*, revealing latent node relations and enabling richer node representation through an extended graph learning-convolutional technique.

### Molecule graph representation

**Fig. 3:**
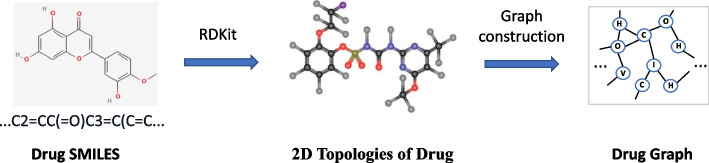
The procedure of constructing a drug molecule graph. The SMILES representations are utilized for molecule graph construction. Due to the complexity of the drug graph, we have depicted only a portion of the full graph for clarity and ease of demonstration

It can be seen from the Fig. 3 that the molecule graph constructed for extracting the small-molecule representation is the same as that of GraphDTA [45] and DGraphDTA [48]. But for the feature extraction, we use graph learning-convolutional networks, instead of conventional GCN or GAT, to get a richer representation.

Specifically, molecules are commonly depicted using SMILES [25] string, transforming atoms and covalent bonds into sequences of ASCII characters. Conveniently, this format can be reverted to molecular structures using prevalent molecular processing software like RDKit [49]. Subsequently, a molecular graph is formed, representing atoms as nodes and chemical bonds as edges. To elaborate, in the creation of the molecular graph, each atom within the molecule is designated as a node in the graph. An edge is then introduced between two atom nodes if they share a bond. This construction method is illustrated in Fig. 3. Besides, a self-loop is incorporated into the molecular graph, connecting each atom node to itself for better aggregating information of the drug molecule.

Beyond just nodes and edges, it’s crucial to define the features of each atom node to ensure they are distinguishable. This is because atoms, the smallest units in chemical reactions, exhibit varied chemical properties due to differences in size and charge. The selected molecular atom node features are the same as those in GraphDTA [45] and WGNN-DTA [50], which is illustrated in Table 1. By detailing the specific features of different atom nodes, the chemical and binding characteristics of small molecules can be more thoroughly represented. Incorporating these factors that influence molecular binding is key to enhancing prediction performance.

Following the molecular graph’s construction, GLCN-DTA utilizes a two-layer graph learning-convolutional network for extracting drug graph features. Graph learning-convolutional networks will be elaborated in the subsequent section. A global pooling process also is employed for the extracted representation. Molecules with different sizes are extracted into latent vectors with the same size for the following performing DTA prediction.

**Table 1:** Atom node feature for molecule graph

Feature name	Feature description	Dimension
Atom type	One-hot encoding of the atom	44
Atom neighbors	One-hot encoding of the degree of the atom in the molecule, which is the number of directly-bonded neighbors	11
Number of hydrogens	One-hot encoding of the total number of $\textrm{H}$ bound to the atom	11
Number of implicit hydrogens	One-hot encoding of the number of implicit $\textrm{H}$ bound to the atom	11
Aromatic flag	Whether the atom is aromatic	1
All	All features of the atom	78

### Protein graph representation

Similarly, for the process of the protein graph, the contact map is first predicted from the protein sequence, and a protein graph is then constructed based on it, basically the same as that of DGraphDTA [48]. But for feature extraction and learning, we are also different from existing graph-based methods in that GLCN-DTA can learn robust features with graph learning operation instead of using a fixed threshold-based adjacent matrix.

**Fig. 4:**
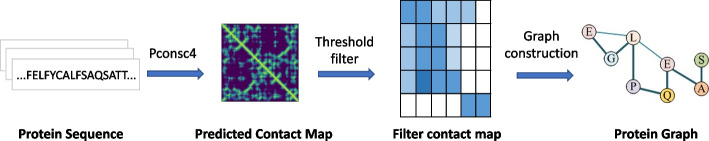
The procedure of constructing a protein graph. The protein sequence are utilized for protein graph construction

Specifically, in a manner akin to drug molecule processing, the first step in extracting protein representation is generating the protein graph. This is followed by feature extraction using a GLCN on this graph, a process illustrated in Fig. 4.

Protein structure prediction aims to analyze and assemble the three-dimensional structure of a protein based on its sequence. This structural data encompasses the angles and distances between various residue pairs. A contact map, often an output of structure prediction techniques, typically takes the form of a matrix. This contact map effectively represents the protein’s high-dimensional structure and is key to identifying interactions among protein residues. Typically, residues are deemed to be in contact if the Euclidean distance between any two atoms is below a certain threshold [59]. In practice, Pconsc4 is used to predict the contact map, which is a fast, simple, open-source, and efficient method. Pconsc4 outputs the likelihood of contact between residue pairs, and applying a 0.5 threshold yields a contact map shaped (*N*, *N*), where *N* represents the count of nodes (residues). This result effectively serves as the protein’s adjacency matrix. However, using the hard threshold to get a fixed adjacent matrix may be existing noises. So we apply the extended graph learning-convolutional network to learn a soft adjacent matrix that will be introduced in the following section.

In the protein graph that’s been built, residues serve as nodes, and thus it’s essential to define the attributes of these residue nodes. The features selected for the residues are listed in Table 2, consistent with the features identified in DGraphDTA [48]. The features of each residue node are defined by the distinct R functional groups they possess. These node features are represented by attributes such as hydrophobicity, polarity, charge, aromaticity, and so on. Furthermore, Position-Specific Scoring Matrix (PSSM) is a prevalent protein representation in proteomics. In PSSM, scores are assigned to each residue position based on sequence alignment outcomes, serving to characterize the residue node’s features. Consequently, this paper utilizes 54-bit features to describe each residue node, with the specifics of these features presented in Table 2. The dimensions of the node features are thus (*N*, 54). Both the adjacency matrix and the node features are then processed via a graph learning-convolutional network to derive the vector representation of the corresponding protein.

Since the development of the protein graph and its feature set is reliant on sequence alignment outcomes, preparatory procedures including sequence alignment and screening are vital. These preliminary steps are the same as used in DGraphDTA [48]. Specifically, in the operation of Pconsc4 and the computation of PSSM, the protein sequence alignment results serve as the initial input. Therefore, in the pre-processing phase, it’s essential to first align all protein sequences in the benchmark datasets. To enhance computation speed, HHblits [60] is employed for the protein sequence alignment. Following this, the alignment results are processed using HHfilter [60] and CCMPred [61] scripts to obtain alignments formatted in the PSICOV [62] style.

**Table 2:** Residue node feature for protein graph

Number	Feature description	Dimension
1	One-hot encoding of the residue symbol	21
2	Position-specific scoring matrix (PSSM)	21
3	Whether the residue is aliphatic	1
4	Whether the residue is aromatic	1
5	Whether the residue is polar neutral	1
6	Whether the residue is acidic charged	1
7	Whether the residue is basic charged	1
8	Residue weight	1
9	The negative of the logarithm of the dissociation constant for the -COOH group [64]	1
10	The negative of the logarithm of the dissociation constant for the $-\textrm{NH}_3$ group [64]	1
11	The negative of the logarithm of the dissociation constant for any other group in the molecule [64]	1
12	The pH at the isoelectric point [64]	1
13	Hydrophobicity of residue $(\textrm{pH}=2)$ [65]	1
14	Hydrophobicity of residue $(\textrm{pH}=7)$ [66]	1
15	All features of the residue	54

### Graph learning-convolutional network

We incorporate extended graph *learning-convolutional network* (GLCN) inspired by [57, 58] into existing graph architecture to learn a *soft adjacent matrix* ${\textbf{A}}$ to model the graph context for DTA tasks, The detailed GLCN is illustrated in Fig. 2.

Mathematically, a graph ${\mathcal {D}}$ is defined as $G=(V, E)$, where $V=\left\{ v_1,\ldots , v_N \right\}$ represents a set of *N* nodes. The edge set $E\subset V \times V$ comprises edges $e_{ij}=(v_i, v_j) \in E$, each indicating the existence of relation $\alpha _{ij} \in R$ from node $v_i$ to node $v_j$.

#### Graph learning

Given a set of graph nodes input ${\textbf{V}} = [{\varvec{v}}_1,\ldots ,{\varvec{v}}_N]^T \in {\mathbb {R}}^{N \times d_{model}}$, where *N* represents the total number of nodes, and each ${\varvec{v}}_i \in {\mathbb {R}}^{d_{model}}$ denotes the feature of the *i*-th node in the graph. The initial state of ${{\textbf {V}}}^{0}$ corresponds to either the atom node features in the molecule graph or the residue node features in the protein graph. GLCN-DTA initially creates a *soft adjacency matrix* ${\textbf{A}}$ via a graph learning operation, which illustrates the weight of pairwise relationships between nodes. It then employs a multi-layer perceptron (MLP) network, akin to the method in [57], to extract features ${\textbf{H}}$ for each node $v_i$ from the input ${\textbf{V}}$. Following this, a graph convolution operation is applied to the features ${\textbf{H}}$, enabling the propagation and aggregation of information between nodes, ultimately leading to a new feature representation ${\textbf{V}}'$.

Mathematically, a *soft adjacency matrix* ${\textbf{A}}$ is learned using a single-layer neural network, which can be represented as follows:

1 $$\begin{aligned} {\left\{ \begin{array}{ll} {\textbf{A}}_i = \textrm{softmax} ({\textbf{e}}_{i}), \quad i = 1, \dots , N, \quad j = 1, \dots , N,\\ \textrm{e}_{ij} = \textrm{LeakyRelu}({\textbf{w}}_i^T| {\varvec{v}}_i - {\varvec{v}}_j|)), \end{array}\right. } \end{aligned}$$

where ${{\textbf {w}}}_i \in {\mathbb {R}}^{d_{model}}$ represents a learnable weight vector. To address the issue of vanishing gradients during the training phase, the LeakyRelu activation function is utilized instead of the traditional Relu function. The $\textrm{softmax}(\cdot )$ operation is applied to each row of ${{\textbf {A}}}$, ensuring that the learned *soft adjacency matrix* ${{\textbf {A}}}$ adheres the following property:

2 $$\begin{aligned} \sum \nolimits ^N_{j=1} A_{ij} =1, A_{ij}\ge 0. \end{aligned}$$

The optimization of the learnable weight vector ${{\textbf {w}}}_i$ is conducted using a modified loss function, adapted from [58], which can be expressed as follows:

3 $$\begin{aligned} {\mathcal {L}}_{\text {GL}} = \frac{1}{N^2} \sum \nolimits ^N_{i,j=1}\exp (A_{ij} + \eta \Vert {\varvec{v}}_i - {\varvec{v}}_j\Vert ^2_2 ) +\gamma \Vert {{\textbf {A}}}\Vert ^2_F, \end{aligned}$$

where $\Vert \cdot \Vert _F$ denotes the Frobenius norm. Intuitively, the first term implies that when nodes ${\varvec{v}}_i$ and ${\varvec{v}}_j$ are spatially distant in higher-dimensional space, it encourages a smaller weight value $A_{ij}$, with the exponential function amplifying this effect. Conversely, nodes closer in the higher-dimensional space are likely to have stronger connection weights. This approach helps in minimizing the influence of noise nodes during graph convolution. $\eta$ serves as a balancing parameter, determining the significance of nodes within the graph. Additionally, averaging the loss is crucial due to the variable number of nodes across different graphs. The second term focuses on maintaining the sparsity of the *soft adjacency matrix* ${{\textbf {A}}}$, with $\gamma$ as the trade-off parameter. A larger $\gamma$ results in a sparser *soft adjacency matrix* ${{\textbf {A}}}$. The loss function ${\mathcal {L}}_{\text {GL}}$ is incorporated as a regularization term in the final loss equation [Eq. (6)], as suggested in [58], to avoid trivial solutions like ${{\textbf {w}}}_i={{\textbf {0}}}$.

#### Graph convolution

Graph convolutional network (GCN) is utilized to capture global information and the features of nodes from the graph. We perform graph convolution on the node features $v_i$.

Firstly, with ${{\textbf {V}}}^{0} \in {\mathbb {R}}^{N \times d_{model}}$ serving as the initial input layer of the graph, *hidden features* ${{\textbf {h}}}_{ij}^{l}$ between nodes $v_i$ and $v_j$ are extracted from the graph. This extraction process utilizes the *node-node* pairs $(v_i, v_j)$ data in the *l*-th convolution layer, and the computation is performed as follows:

4 $$\begin{aligned} {\varvec{h}}_{ij}^{l} = \sigma ({{\textbf {W}}}^l_{v_{i}h} {{\textbf {v}}}_i^l + {{\textbf {W}}}^l_{v_{j}h} {{\textbf {v}}}_j^l + {{\textbf {b}}}^l ), \end{aligned}$$

where ${{\textbf {W}}}^l_{v_{i}h}$ and ${{\textbf {W}}}^l_{v_{j}h} \in {\mathbb {R}}^{d_{model} \times d_{model}}$ are the learnable weight matrices specific to the *l*-th convolution layer, while ${{\textbf {b}}}^l \in {\mathbb {R}}^{d_{model}}$ serves as a bias parameter. The terms ${{\textbf {v}}}_i^l$ and ${{\textbf {v}}}_j^l$ represent the features of the *i*-th and *j*-th nodes in the *l*-th convolution layer, respectively. The function $\sigma (\cdot )=\max (0,\cdot )$ is a non-linear activation function. The *hidden features* ${\varvec{h}}_{ij}^l \in {\mathbb {R}}^{d_{model}}$ represent the combination of graph features and the relational embedding between nodes $v_i$ and $v_j$, which is vital for compiling a more comprehensive representation for the DTA prediction task.

Next, the *node embedding* ${{\textbf {v}}}_{i}^{l+1}$ aggregates information from the *hidden features* ${\varvec{h}}_{ij}^l$ through graph convolution, thereby updating the node representation. Since the graph learning layer is capable of generating an optimal adaptive graph *soft adjacency matrix* ${{\textbf {A}}}$, the graph convolution layers can acquire task-specific *node embeddings* by applying a layer-wise propagation rule. For node $v_i$, the process can be described as follows:

5 $$\begin{aligned} {{\textbf {v}}}_i^{(l+1)} = \sigma ( {{\textbf {A}}}_i {{\textbf {h}}}_{i}^{l} {{\textbf {W}}}^{l}), \end{aligned}$$

where ${{\textbf {W}}}^l \in {\mathbb {R}}^{d_{model} \times d_{model}}$ represents a layer-specific learnable weight matrix in the *l*-th convolution layer. The term ${{\textbf {v}}}_i^{(l+1)} \in {\mathbb {R}}^{d_{model}}$ signifies the node embedding for node $v_i$ in the $(l+1)$-th convolution layer. After progressing through *L* layers, the contextual information ${{\textbf {v}}}_i^{L}$ is obtained, encompassing global information for every node $v_i$. This information, ${{\textbf {v}}}_i^{L}$, is then carried forward to subsequent steps for the execution of the DTA prediction task.

In practice, the representations of both the molecule graph and the protein graph are input into the GLCN to separately generate drug features and protein features. Following this, a global pooling process is applied to these extracted features. This step is crucial as it standardizes the size of the resultant latent vectors, preparing them for use in subsequent DTA prediction tasks.

### Performing DTA prediction

Following the feature extraction process by GLCN, latent vectors for both the protein and molecular graphs are derived. These two latent vectors are then concatenated and further processed through two fully connected layers to achieve the final binding prediction. One LeakyRelu activation function is after the first fully connected layer. We also employ dropout to avoid overfitting. This process is visually detailed in the right part of Fig. 2.

### Model optimization

The model parameters for the entire network are trained jointly by minimizing the loss function expressed as follows:

6 $$\begin{aligned} {\mathcal {L}}_{\text {total}} = {\mathcal {L}}_{\text {dta}} + \lambda {\mathcal {L}}_{\text {GL}}, \end{aligned}$$

where ${\mathcal {L}}_{\text {GL}}$ is as defined in Eq. 3, with $\lambda$ serving as a balancing parameter. In practical terms, ${\mathcal {L}}_{\text {dta}}$ is computed using the mean squared error (MSE) loss to minimize the discrepancy between the predicted affinity values and the actual (ground truth) values.

## Results and discussion

### Datasets

We conducted experiments using four benchmark datasets, Davis [63], KIBA [64], Metz [65], and ToxCast [66], for training our model and evaluating its performance. Davis [63] and KIBA [64] datasets are widely recognized benchmarks for DTA prediction and have been previously used in DeepDTA [34]. They are also publicly available. To more thoroughly assess our model’s performance, we have incorporated the Metz [65] and ToxCast [66] datasets into this study, which include a variety of different types of targets, encompassing both kinases and non-kinases. Distinct from the previously mentioned benchmark datasets, the Metz and ToxCast datasets measure the binding affinities of drug–target pairs using inhibition constant ($K_i$) and concentration for 50% of maximal effect ($EC_{50}$), respectively. Detailed information about these four datasets can be found in Table 3.

The Davis [63] dataset was compiled by choosing specific kinase proteins and their inhibitors, where the binding affinity is indicated by the dissociation constant $K_d$. This dataset encompasses 442 proteins, 68 drugs, and a total of 30,056 drug–target interactions. The average length of the drug SMILES strings is 64, while the average length of the protein sequences is 788. The processing of affinity values in this dataset follows the same methodology as used in DeepDTA [34], employing the following equation:

7 $$\begin{aligned} p K_d=-\log _{10} \frac{K_d}{10^9} \end{aligned}$$

The KIBA [64] dataset compiles kinase inhibitor biological activities from multiple sources, including the inhibition constant ($K_i$), dissociation constant ($K_d$), and the half-maximal inhibitory concentration ($IC_{50}$). It utilizes the KIBA score to predict biological activity. This dataset features 229 proteins, 2111 drugs, and a total of 118,254 drug–target interactions. The average length of the drug SMILES strings in this dataset is 58, and the average protein sequence length is 728.

In the benchmark setup, each dataset is partitioned into various parts, with one part designated for testing and the remaining parts used for cross-training and validation. By conducting tests on these two datasets, the predictive capability of the method can be thoroughly assessed.

**Table 3:** Summary of the benchmark datasets

Dataset	Proteins	Drugs	Binding entries	Train	Test
Davis	442	68	30,056	25,046	5010
KIBA	229	2111	118,254	98,545	19,709
Metz	170	1423	35,259	28,207	7052
ToxCast	37	3098	114,626	80,251	34,375

### Evaluation metrics

DTA prediction is treated as a regression problem, and our model’s performance was evaluated using three metrics: mean squared error (MSE), concordance index (CI), and regression toward the mean (denoted as $r^2_m$ index).

MSE measures the deviation between predicted and actual values using a squared loss function, which can be expressed as:

8 $$\begin{aligned} M S E=\frac{1}{n} \sum _{i=1}^n\left( {\hat{y}}_i-y_i\right) ^2 , \end{aligned}$$

where ${\hat{y}}_i$ represents the predicted value, $y_i$ is the actual (true) value, and *n* denotes the total number of drug–target pairs in the dataset.

CI is employed to assess whether the predicted DTA values for two randomly selected drug–target pairs maintain the same rank order as their actual values. This measure is important for evaluating the model’s ability to accurately rank the interactions in terms of their binding affinities, which can be expressed as:

9 $$\begin{aligned} C I=\frac{1}{Z} \sum _{d_x>d_y} h\left( b_x-b_y\right) , \end{aligned}$$

10 $$\begin{aligned} h(x)= {\left\{ \begin{array}{ll}1, &{} \text{ if } x>0 \\ 0.5, &{} \text{ if } x=0 \\ 0, &{} \text{ if } x<0\end{array}\right. } , \end{aligned}$$

where $b_x$ represents the predicted value corresponding to the larger affinity $d_x$, and $b_y$ denotes the predicted value for the smaller affinity $d_y$. The function *h*(*x*) is a step function used in the calculation. The term *Z* serves as a normalization constant, signifying the total number of drug–target pairs involved in the analysis.

$r^2_m$ metric is utilized to assess the external predictive capability of the model. This evaluation is conducted as follows:

11 $$\begin{aligned} r_m^2=r^2 \times \left( 1-\sqrt{r^2-r_0^2}\right) , \end{aligned}$$

where $r^2$ represents the squared correlation coefficient between the true and predicted values with intercepts. Conversely, $r^2_0$ denotes the squared correlation coefficient for the true and predicted values without intercepts.

### The setting of the hyperparameters

Our methods were executed using PyTorch, with the model trained on mini-batches of size 128. We employed an initial learning rate of 0.0005, complemented by a warm setting. Additionally, we utilized learning rate decay, reducing it by 20% every 40 epochs. The Adam optimizer was the choice for optimization. The training spanned over 1000 epochs. Detailed information on the training hyperparameters for our model is listed in Table 4.

**Table 4:** Hyperparameters used in our experiments

Hyperparameter	Setting
Learning rate	0.0005
Epoch	1000
Batch size	128
Optimizer	Adam
GLCN layers	2
Fully connected layers after concatenation	2
dropout rate	0.2
$d_{model}$	128
$\lambda$	1
$\eta$	1
$\gamma$	1

**Table 5:** Prediction performance on the Davis dataset

Model	MSE	Cl	$r_m^2$
KronRLS [67]	0.379	0.871	0.407
SimBoost [68]	0.282	0.872	0.644
DeepDTA [34]	0.261	0.878	0.630
WideDTA [35]	0.262	0.886	0.633
MATT_DTI [69]	0.229	0.890	0.682
DeepGS [70]	0.252	0.882	0.686
AttentionDTA [71]	0.245	0.887	0.657
GraphDTA [45]	0.229	0.893	0.649
DeepGLSTM [72]	0.232	0.895	0.680
SubMDTA [73]	0.218	0.894	0.719
DGraphDTA^a^ [48]	0.240	0.890	0.659
GEFA] [52]	0.228	0.893	–
STAMP-DPI [53]	0.474	–	–
WGNN-DTA^a^ [50]	0.215	0.900	0.711
GLCN-DTA (ours)	0.215	0.903	0.720

*MSE* mean squared error, *CI* concordance index. The $r^2_m$ index was used in DeepDTA [34], which can be used to evaluate the external predictive performance of quantitative structure-activity relationship models

^a^The experimental results are acquired from [5]

**Table 6:** Prediction performance on the KIBA dataset

Model	MSE	Cl	$r_m^2$
KronRLS [67]	0.411	0.782	0.342
SimBoost [68]	0.222	0.836	0.629
DeepDTA [34]	0.194	0.863	0.673
WideDTA [35]	0.179	0.875	0.675
MATT_DTI [69]	0.150	0.889	0.756
DeepGS [70]	0.193	0.860	0.684
AttentionDTA [71]	0.162	0.882	0.735
GraphDTA [45]	0.147	0.889	0.674
DeepGLSTM [72]	0.133	0.897	0.792
SubMDTA [73]	0.129	0.898	0.793
DGraphDTA^a^ [48]	0.147	0.891	0.767
WGNN-DTA^a^ [50]	0.140	0.899	0.749
GLCN-DTA (ours)	0.127	0.899	0.792

*MSE* mean squared error, *CI* concordance index. The $r^2_m$ index was used in DeepDTA [34], which can be used to evaluate the external predictive performance of quantitative structure-activity relationship models

^a^The experimental results are acquired from [5]

### Comparison with existing methods

To thoroughly assess our method’s efficacy in DTA prediction, we benchmarked it against existing methods such as DeepDTA [34], GraphDTA [45], DGraphDTA [48], and others. This comparison utilized the same benchmark datasets, Davis [63], KIBA [64], Metz [65], and ToxCast [66], and implemented identical training and test sets. Additionally, uniform performance measures were employed for evaluation. The results from other methods were sourced from their respective publications. Performance comparisons are presented in Tables 5 and 6, where lower MSE, or higher CI and $r^2_m$ scores, indicate superior model performance.

Specifically, Table 5 displays the performance metrics (MSE, CI, and $r^2_m$) of various models on the Davis dataset. Our method markedly outperformed others on this dataset, achieving an MSE of 0.215, a CI of 0.903, and an $r^2_m$ of 0.720. These results represent improvements of + 0.3%, + 0.3%, and + 0.1%, respectively, over the previously best-performing method.

Additionally, we assessed our model on the KIBA dataset. As indicated in Table 6, GLCN-DTA excelled among existing methods, achieving an MSE of 0.127, which is + 0.2% higher than the prior best method. The CI performance matched that of the leading WGNN-DTA method. In terms of $r^2_m$, GLCN-DTA was very close to the top-performing SUbMDTA, differing by only 0.001. These outcomes suggest that our proposed method is effective for DTA prediction.

**Table 7:** Prediction performance on the Metz and ToxCast datasets

Model	Metz			ToxCast		
	MSE	Cl	$r_m^2$	MSE	Cl	$r_m^2$
GraphDTA [45]	0.282	0.816	0.681	0.215	0.843	0.330
MGraphDTA [74]	0.265	0.822	0.701	0.176	0.902	0.430
GPCNDTA [11]	0.248	0.834	0.686	0.165	0.904	0.474
GLCN-DTA (ours)	0.236	0.848	0.713	0.152	0.917	0.492

We also report the performance of GLCN-DTA on the Metz and ToxCast test sets to verify the effectiveness of GLCN-DTA on a vast majority of different types of targets, with MSE, CI, and $r^2_m$ values of 0.236, 0.848, and 0.713 for Metz, and 0.152, 0.917, and 0.492 for ToxCast, respectively, as detailed in Table 7. In terms of prediction accuracy, GLCN-DTA demonstrates a notable advantage over GraphDTA [45], MGraphDTA [74], and GPCNDTA [11] on both the Metz and ToxCast datasets, which reveals the strong effectiveness of GLCN-DTA.

The superiority of our model can be attributed to our used extended graph learning-convolutional network. To derive more distinctive molecular and protein representations, we employed the soft adjacency matrix, which aggregates complex information from molecules and protein graphs via graph learning and graph convolution operations. Consequently, our model integrates the intrinsic information of complex compounds and protein graphs into a more comprehensive representation, enhancing both the accuracy and robustness of the model.

### Ablation studies

We conducted a range of ablation studies to examine the effects of various hyper-parameters on the model’s DTA prediction performance. For these studies, all models were trained from scratch using the default standard settings.

#### Ablation study for the number of GLCN layers

We conducted an ablation study on the Davis dataset to evaluate how the number of GLCN layers affects DTA prediction performance. As indicated in Table 8, the optimal results were consistently achieved with a 2-layer model, rather than with models having 1, 3, or 4 layers. This outcome highlights a known aspect of GCN [43] models: the deeper the model (i.e., the greater the number of layers), the higher the likelihood of overfitting. Therefore, in practical applications, it’s advisable to set a specific number of graph layers to optimize performance.

**Table 8:** Ablation study for the number of GLCN layers on the Davis dataset with GLCN-DTA methods

The number of GLCN layers	MSE	Cl	$r_m^2$
1	0.232	0.885	0.700
2	0.215	0.903	0.720
3	0.226	0.893	0.713
4	0.248	0.876	0.692

#### Ablation study for the graph learning module

As indicated in Table 9, the removal of the graph learning component from GLCN-DTA results in a significant decrease in performance metrics on both Davis and KIBA datasets, particularly on the more complex, longer sequences of the KIBA dataset. This suggests that the graph learning element plays a crucial role in handling complex structures in datasets, enhancing generalization, and learning superior representations for improved DTA prediction tasks.

**Table 9:** Ablation study for the graph learning module with GLCN-DTA methods

Model	Davis			KIBA		
	MSE	Cl	$r_m^2$	MSE	Cl	$r_m^2$
GLCN-DTA	0.215	0.903	0.720	0.127	0.899	0.792
w/o graph learning	$\downarrow$0.03	$\downarrow$0.02	$\downarrow$0.05	$\downarrow$0.05	$\downarrow$0.04	$\downarrow$0.08

#### Ablation study for the weight of graph learning loss

We conducted an ablation study focused on a key hyper-parameter: the trade-off ratio $\lambda$, which is the weight of the graph learning loss in Eq. 6. Specifically, we carried out a series of experiments varying the $\lambda$ value to assess its impact on DTA prediction performance. The results, reported in Table 10, explore $\lambda$ values within the range [0, 0.5, 1, 2, 4]. It was observed that our method performed optimally at $\lambda = 1$, surpassing other configurations. Therefore, in practical applications, setting a task-specific value for $\lambda$ is crucial to ensure the best possible results.

**Table 10:** Ablation study for the weight of graph learning loss on the Davis dataset with GLCN-DTA methods

The weight of the graph learning $\lambda$	MSE	Cl	$r_m^2$
0	0.245	0.883	0.670
0.5	0.215	0.903	0.720
1	0.227	0.894	0.712
2	0.238	0.890	0.685
4	0.241	0.885	0.679

**Fig. 5:**
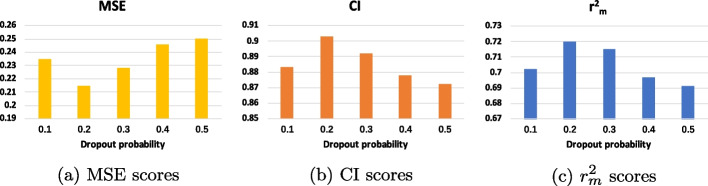
Ablation study for various dropout probabilities on the Davis datasets with GLCN-DTA methods

#### Ablation study for various dropout probabilities

In the stage of performing DTA prediction, the latent vectors representing protein and drug features are concatenated and then passed through two fully connected layers to determine the final binding prediction. To mitigate overfitting, dropout is applied after each fully connected layer. During forward propagation, dropout randomly deactivates neurons with a probability p, enhancing the model’s generalization and effectively addressing overfitting. To assess the impact of varying dropout probabilities, we conducted tests on the Davis dataset with different values of p. The results, depicted in Fig. 5, indicate that the optimal dropout probability is 0.2, as evidenced by the lowest MSE value and the best CI and $r^2_m$ performances at this setting. Excessively high dropout probabilities can cause underfitting and ineffective extraction of protein features, while overly low probabilities might not sufficiently prevent overfitting. Therefore, an appropriately balanced dropout probability is essential for achieving the best predictive performance.

## Conclusions

To enhance DTA prediction capabilities, this paper introduces the GLCN-DTA method, effectively characterizing small molecules and protein sequences through the construction of molecular graphs and protein graphs based on contact maps. Utilizing the expanded capabilities of GLCN for advanced feature extraction, the resulting latent vectors provide a more comprehensive representation of proteins and molecules. Extensive experimental results show that GLCN-DTA is not only applicable to DTA prediction but also excels in processing complex and longer datasets. This makes it a robust tool for virtual screening of target proteins and aiding in the discovery of lead compounds.

However, our graph-based DTA prediction approach does not currently account for protein classification or distinguish between structurally ordered and intrinsically disordered proteins. Similar to other graph-based methods [48, 50, 52, 53], it just concentrates on enhancing the extraction of drug and target representations and feature fusion, and subsequent affinity prediction. This specificity might restrict its applicability scope in contexts involving intrinsically disordered proteins and be more suitable for scenarios with structurally ordered proteins. Moreover, our model solely provides predictions for binding affinity without considering the specific binding cavities, lacking the intuitive interpretability of the model. In our future work, we aim to incorporate domain knowledge such as the classification of proteins into structurally ordered protein and intrinsically disordered protein categories into the model, to improve DTA prediction. We also plan to incorporate binding sites as prior knowledge during training to enhance the interpretability of the model. Efforts are underway to organize relevant datasets for these enhancements. Additionally, we intend to integrate additional drug characteristics, such as textual descriptions of proteins and drugs, into the model. This integration aims to further enhance the performance of drug–target binding prediction models by incorporating diverse aspects of drug information.

## Data Availability

The Davis, KIBA, Metz, and ToxCast datasets used in the current study can be downloaded from https://github.com/LiZhang30/GPCNDTA.
